# NAD^+^-Consuming Enzymes in Stem Cell Homeostasis

**DOI:** 10.1155/2023/4985726

**Published:** 2023-02-08

**Authors:** Xiuna Ji, Mingyue Zheng, Tao Yu, Jie Kang, Tingjun Fan, Bin Xu

**Affiliations:** ^1^College of Marine Life Sciences, Ocean University of China, Qingdao, China; ^2^Guangdong Provincial People's Hospital, Guangdong Academy of Medical Sciences, Guangzhou, China; ^3^Affiliated Hospital of Qingdao University, Qingdao, China

## Abstract

Nicotinamide adenine dinucleotide (NAD^+^) is a coenzyme used in redox reactions, energy metabolism, and mitochondrial biogenesis. NAD^+^ is also required as a cofactor by nonredox NAD^+^-dependent enzymes. Hundreds of enzymes that consume NAD^+^ have been identified. The NAD^+^-consuming enzymes are involved in a variety of cellular processes such as signal transduction, DNA repair, cellular senescence, and stem cell (SC) homeostasis. In this review, we discussed how different types of NAD^+^-consuming enzymes regulate SC functions and summarized current research on the roles of the NAD^+^ consumers in SC homeostasis. We hope to provide a more global and integrative insight to the mechanism and intervention of SC homeostasis *via* the regulation of the NAD^+^-consuming enzymes.

## 1. Introduction

Nicotinamide adenine dinucleotide (NAD^+^) has been described as central coenzyme for redox reactions [1]. During the oxidation of glucose and fatty acids, NAD^+^ is reduced to NADH, which acts as a ubiquitous cellular electron donor. Two NADH are generated by glycolysis and converted back to NAD^+^ [2]. In addition to energy metabolism, NAD^+^ is used as a cofactor or cosubstrate by hundreds of enzymes [3]. This net consumption of NAD^+^ is compensated by *de novo* starting with tryptophan, or from salvage pathways starting with NAD^+^ precursors, thus maintaining a balanced pool under normal physiological conditions [2].

**Figure 1:**
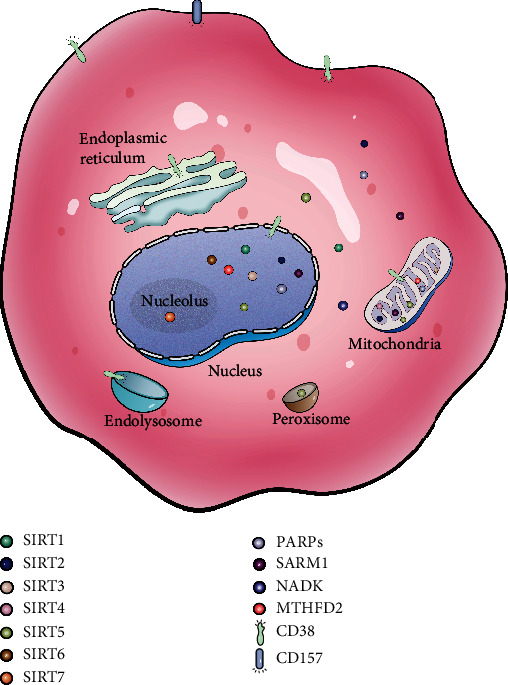
Locations of NAD^+^-consuming enzymes. SIRT1 is reported to locate in both in nucleus and cytosol. SIRT2, predominantly resided in cytosol, may also exist in mitochondria and nucleus. SIRT3 is a major mitochondrial deacetylase and also plays a role in nucleus. SIRT4 is only found in mitochondria. SIRT5 locates in mitochondrial matrix and intermembrane space, cytosol, peroxisome, and nucleus. SIRT6 is only found in nucleus. SIRT7 is the only sirtuin protein that mainly locates in nucleolus. PARPs reside in cytosol, mitochondria, and nucleus. CD38 is observed both in plasma membrane (catalytic domain facing outside or inside) and intracellular membranes (including endoplasmic reticulum, nucleus, mitochondria, and endolysosome). CD157 is a glycophosphatidylinositol-anchored protein with the catalytic domain facing outside. Sterile alpha and Toll/interleukin receptor (TIR) motif-containing 1 (SARM1) locates in cytosol, mitochondria, and nucleus. Methylenetetrahydrofolate dehydrogenase 2 (MTHFD2) is found in mitochondria and nucleus. NAD^+^ kinase (NADK) only resides in cytosol.

**Figure 2:**
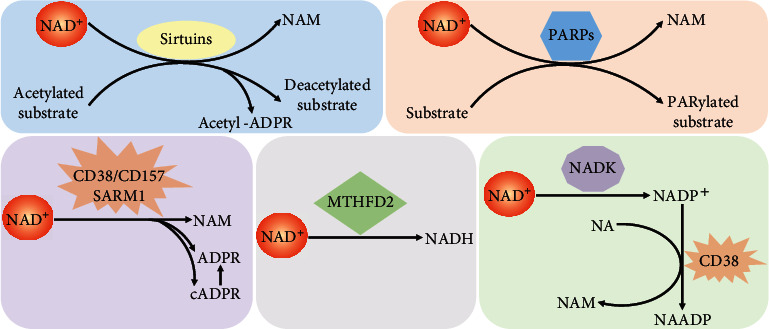
Catalytic reactions of NAD^+^-consuming enzymes using NAD^+^ as a substrate. Sirtuins remove acyl groups from lysine residues on target substrates including proteins and lipids. ADP-ribose (ADPR) cleaved from NAD^+^ serves as an acyl group acceptor to generate acetyl-ADPR. PARPs transfer the ADP-ribose from NAD^+^ to proteins, DNA, and RNA (known as PARylation). CD38, CD157, and sterile alpha and Toll/interleukin receptor (TIR) motif-containing 1 (SARM1) are multifunctional ectoenzymes with both glycohydrolase and ADP-ribosyl cyclase activities. Their main catalytic activity is the hydrolysis of NAD^+^ to NAM and ADP-ribose (ADPR). They also catalyze NAD^+^ to NAM and cADPR. Methylenetetrahydrofolate dehydrogenase 2 (MTHFD2) uses NAD^+^ to generate NADH. NAD^+^ can also be consumed by NAD^+^ kinase (NADK) to increase NADP^+^ production. Then NADP^+^ is catalyzed to NAADP by CD38.

The diverse enzymes consuming NAD^+^ are found in almost all eukaryotic cells (Figure 1). They have multiple roles in the regulation of cellular processes and functions such as DNA repair, epigenetic modification, and inflammation [4]. These enzymes can be divided into two categories involved in a variety of cellular processes, including stress response, mitochondrial homeostasis, and calcium signaling [5]. The first category breaks down NAD^+^ and transfers the adenosine diphosphate- (ADP-) ribose units to fundamental biomolecules. The second hydrolyzes NAD^+^ to ADP-ribose (ADPR), cyclic ADPR (cADPR), and nicotinamide (NAM), which are dominated by multifunctional ectoenzymes with both glycohydrolase and ADP-ribosyl cyclase activities (Figure 2) [6, 7]. Recent reports show that the above NAD^+^ consumers attracted more attentions in the field of stem cell (SC) homeostasis mechanism and intervention [8]. Therefore, in this review, we focus on the current advances in the roles of NAD^+^-consuming enzymes in SC homeostasis.

The literature search produced a total of 49,519 records (39,570 from Web of Science and 9,949 from PubMed). After title/abstract/language/literature evaluation screening, 99 articles were assessed for full-text eligibility, an additional 22 studies identified through review articles, and a final total of 121 articles were included in the final review (Figure S1).

## 2. Category I: Deacylases (Sirtuins (SIRTs) and Poly(ADP-Ribose) Polymerases (PARPs))

### 2.1. Sirtuins

There are seven sirtuins (SIRT1–7) with a conserved NAD^+^-binding domain in mammals [9, 10]. Sirtuins have various activities, containing different lysine deacetylation reactions, ADP ribosylation, and removal of lipid modifications [11, 12]. They have been found to play roles in multiple cellular functions, such as genomic stability, transcription, signal transduction, and metabolism [13]. Recent studies report that they also directly or indirectly participate in the regulation of multiple signaling pathways to maintain SC homeostasis (Table 1).

#### 2.1.1. SIRT1 (Metabolome-Epigenome Crosstalker)

SIRT1, located both in the nucleus and cytosol, is the largest one in molecular mass and the most extensively studied sirtuin protein [14]. It has been reported to play a crucial role in the SC self-renewal. SIRT1 does not only mediate mouse embryonic SC (mESC) maintenance and embryonic development through deacetylation of methionine adenosyltransferase 2a (MAT2a) [15] but, also modulate mESC differentiation *via* c-Myc-SMPDL3B signaling cascades [16].

There is growing evidence that decreasing intracellular NAD^+^ level and SIRT1 activity are associated with SC senescence *in vivo*. The reduction of cellular NAD^+^ pools blunts the adaptive mitochondrial unfolded protein response (UPR^mt^) pathway, ultimately leading to a loss of mitochondrial homeostasis in a SIRT1-dependent manner. Mitochondrial dysfunction is a biomarker of muscle SC (MuSC) senescence that reduces SC cell number and self-renewal capacity [8]. Moreover, cell culture expansion *in vitro* induces replicative senescence and loss of NAD^+^ homeostasis in hMSCs which correlates with the decreasing of the SIRT1 signaling activity [17]. Similarly, nicotinamide phosphoribosyltransferase (NAMPT), known as a rate-limiting enzyme in the NAD^+^ salvage pathway, suppresses rat MSC senescence *via* NAD^+^-SIRT1 signaling [18]. Above results demonstrate that NAD^+^-SIRT1 axis dysfunction might be a potential checkpoint for stemness loss and homeostasis disruption of SCs.

Furthermore, the NAD^+^-dependent SIRT1 switches metabolic signaling into epigenetics regulation by decreasing H4K16 acetylation and inactivation of muscle gene transcription in MuSCs [14]. SIRT1 also regulates neural SC (NSC) fates by modulating the circadian clock possibly through FOXO3a deacetylation and acts as a gatekeeper of NSCs by responding to metabolic stress [19]. Similarly, SIRT1 also plays an important role in the maintenance of cancer stem cell (CSC) self-renewal. SIRT1 deacetylates *β*-catenin to trigger its concentration in the nucleus. The nuclear *β*-catenin further promotes the transcription of *NANOG*, which aids in the maintenance of liver CSC self-renewal [20]. SOX2 is also a key downstream regulator of SIRT1-mediated liver CSC self-renewal and tumorigenicity. SIRT1 controls the *SOX2* gene transcription through chromatin-based epigenetic modification that is reliant on DNA methylation [21]. These results imply its important regulatory roles in the metabolome-epigenome signaling cascade in SC homeostasis.

#### 2.1.2. SIRT2 (Major Cytosolic Sirtuin)

SIRT2 is the only sirtuin protein predominantly resided in the cytosol, although it has also been found in the mitochondria and nucleus [22, 23]. It is identified as a direct regulator of PAX7 acetylation and asymmetric division in MuSCs [24]. SIRT2 also prevents mitochondrial stress-induced hematopoietic SC (HSC) death by repressing NLRP3 inflammasome and caspase 1 activation [25]. In addition, SIRT2 inhibits the activities of four glycolytic enzymes (ALDOA, GAPDH, PGK1, and ENO1) by regulating their acetylation status, thereby interfering the metabolic state during somatic reprogramming of induced pluripotent SCs (iPSCs) [26]. These findings suggest that SIRT2 might be another key enzyme in SC homeostasis, involved in transcriptional activity, inflammatory response, and metabolic switch.

#### 2.1.3. SIRT3 (Dual Function for Mitochondrial Homeostasis and Genomic Stability)

SIRT3 is a major mitochondrial deacetylase, regulating mitochondrial metabolism [27], also reported to have a role in deacetylating histones in the nucleus [28]. It is the most abundant sirtuin family member in HSCs for their homeostasis [29]. SIRT3 cooperates with autophagy by promoting its expression to decelerate hematopoietic aging, and positive intervention to the autophagy-SIRT3 axis leads to blood rejuvenation [30]. Besides, SIRT3 decreases in human MSCs with *in vitro* passaging; its knockdown accelerates aging and inhibits efficient differentiation of MSCs into adipocytes and osteoblasts; instead, SIRT3 overexpression restores their differentiation capacity and reduces oxidative stress in later-passage MSCs [31]. The mechanisms involved may contribute to that SIRT3 helps to activate antioxidant enzymes (i.e., CAT and MnSOD) [32] and mitophagy inducing by enhanced mitochondrial ROS [33] and stabilize heterochromatin to counteract MSC senescence by interacting with nuclear envelope proteins and heterochromatin-associated proteins [34]. SIRT3 is also related to regulating mitochondrial quality and function for cellular adaption to hypoxia in rat MSCs [35], promoting *β* cell maturation of mouse ES cells through tricarboxylic acid cycle [36], and ameliorating microglia activation-induced oxidative stress injury through mitochondrial apoptosis pathway in mouse NSCs [37]. A recent research has found that aerobic respiration is upregulated in spermatogonial stem cell (SSC) differentiation in a SIRT3-dependent manner [38, 39]. Above reports show that SIRT3 might regulate SC behavior through multiple pathways, including mitochondrial homeostasis and genomic stability.

#### 2.1.4. SIRT4 (Negative Regulatory Switch)

SIRT4 plays a critical role in cellular metabolism and DNA damage responses in mitochondria [40]. Compared with the other mitochondrial sirtuins, the enzymatic activity of SIRT4 is poorly understood in SCs [12]. Its overexpression triggers senescence in trophoblast SCs (TSCs) due to redox imbalance [41]. Loss of SIRT4 promotes the self-renewal of breast CSCs [42]. SIRT4 appears to exhibit a negative regulatory effect on SC homeostasis, while it remains fundamentally unexplored.

#### 2.1.5. SIRT5 (the Most Widely Functional Sirtuin?)

SIRT5 is a mitochondrial sirtuin localized to the mitochondrial matrix [43]. Interestingly, its target proteins have been found in the mitochondrial matrix and intermembrane space [44], cytosol [45], peroxisome [46], and nucleus [47]. Successive passages *in vitro* cause SIRT5 accumulation in adipose-derived mesenchymal SCs (ADMSCs) with loss of stemness, eventually accelerating cell senescence. SIRT5-deficient ADMSCs exhibit a higher proliferation rate, delayed senescence, decreasing ROS accumulation, and elevating aerobic glycolysis and attenuating mitochondrial respiration in a more endogenous metabolic pattern [48]. In contrast, SIRT5 has also been reported to have positive effects on oxidative phosphorylation [49, 50]. Considering oxidative phosphorylation as the major source of ROS, it is quite surprising that SIRT5-deficient murine embryonic fibroblasts (MEFs) show higher ROS levels [50]. Although SIRT5 function is not well defined, it may be a potential target to promote the self-renewal capacities and maintain the physiological functions of SCs due to its multiple cellular locations [51].

#### 2.1.6. SIRT6 (Specific Switch of Histone H3)

SIRT6, only found in the nucleus, specifically modifies histone H3 to regulate several fundamental processes about lifespan [52]. SIRT6-deficient HSCs exhibited impaired self-renewal ability [53], and its roles in maintaining SC homeostasis and human development might be related to modification of histone H3. Besides, its chromatin remodeling activity is a critical modulator of human development, regulating the transition from pluripotency to differentiated state [54–57]. Human iPSCs derived from SIRT6 Asp63His (D63H) mutant fail to differentiate into embryoid bodies (EBs), functional cardiomyocytes, and neural progenitor cells (NPCs) because of sustained pluripotent gene expression [54]. However, it has been reported to maintain MSC homeostasis by serving as a NRF2 coactivator to transactivate the NRF2-driven antioxidant genes [58]. SIRT6 can also regulate HSC homeostasis through the transcriptional repression of Wnt target genes. Therefore, the function of SIRT6 in maintaining SC homeostasis may not be limited to the nucleus.

#### 2.1.7. SIRT7 (Specific Nucleolar Sirtuin)

SIRT7 is the only sirtuin protein that mainly localizes to the nucleolus [59], involving in ribosomal biogenesis [60], stress responses [61], and senescence [62]. In HSCs, SIRT7 interacts NRF1 to regulate cellular energy metabolism and proliferation through the UPR^mt^. In addition, SIRT7 upregulation improved the regenerative capacity of aged HSCs [63] and hMSCs [64]. Mechanistically, SIRT7 forms a complex with nuclear lamins and heterochromatin proteins to maintain the repressive state of heterochromatin [64]. It also can activate quiescent hair follicle SCs (HFSCs) to initiate the cell cycle by destabilizing NFATc1 [65] and regulate MSC osteogenic differentiation partly by activating Wnt/*β*-catenin signaling [66]. Because of its special location, SIRT7 may play a special role in the regulation of SC homeostasis, which is worthy of further study.

Some sirtuins are detected in multiple cellular compartments, and to shuttle among the various cellular localizations [67]. Different sirtuins have complementary functions and extensive crosstalk to maintain SC homeostasis, besides their own distinct functions. For instance, mitochondrial sirtuins are able to manage these delicate processes accurately by crosstalk between the mitochondria and nucleus [12]. Yuan et al. reported that the reducing expressions of SIRT1 and SIRT3 fail to regulate mitochondrial fitness, DNA repair, and other aging-associated pathways during hMSC culture expansion [17]. SIRT4 and SIRT1 have an inverse relationship in breast CSCs, and SIRT4 inhibits SIRT1 expression by suppressing glutamine metabolism [42]. However, their cooperative relationship and mechanisms are still unclear and poorly understood, and more in-depth studies are needed [12].

### 2.2. PARPs

PARPs family consisted of 17 proteins, widely distributed in all human tissues, and involved in a variety of cellular functions, such as the cellular response to DNA damage and the regulation of gene transcription [68, 69]. PARPs transfer the ADP-ribose from NAD^+^ to fundamental biomolecules (known as PARylation) containing proteins, DNA, and RNA [11, 70–73]. Recently, PARP research has been enriched by the discovery of novel PARP1 interaction partners that regulate its enzymatic activity [74, 75]. It shows that PARP1 contributes to pluripotency, lineage specific differentiation, and reprogramming in various SCs [76].

PARP1 is a component of the groucho/TLE-corepressor complex, which mediates dismissal of the corepressor complex from HES1-regulated promoters during neural stem/progenitor cell (NSC) differentiation [77]. During this process, PARP1 mediates histone H1 eviction from the chromatin fiber [78]. Similarly, PARP1 interacts with PARylates SOX2 directly, in which it may be required for dissociation and degradation of inhibitory SOX2 proteins from the FGF4 enhancer during ESC differentiation [79]. PARP1 can also dominate NSC proliferation by modulating platelet-derived growth factor receptor *α* (PDGFR*α*) expression [80]. PARylation is reported to promote the proliferation and self-renewal of mouse brain NSCs by inhibiting p53 activation [81]. PARP1 loss leads to defects in brain development, increased neuronal density at birth in mice, and enhanced embryonic NSC adhesion to N-cadherin *in vitro* [82].

These results demonstrate the chromatin-related function of PARP1, which PARylates different transcription factors to modulate their DNA binding and transcriptional activity, thereby regulating SC homeostasis [83]. Therefore, site-specific PARylation to drive cell fate may be a very promising approach in SC therapy. Remarkably, CSCs have an increased DNA damage response, and PARP1 is upregulated due to its crucial involvement in DNA repair [84, 85]. It implies that PARP inhibitors can be employed as therapeutic strategies to target CSCs, such as FDA-approved olaparib and rucaparib.

## 3. Category II: NAD^+^ Glycohydrolases (Also Referred to as NADases, including CD38, CD157, and Sterile Alpha and Toll/Interleukin Receptor (TIR) Motif-Containing 1 (SARM1))

### 3.1. CD38

CD38 is considered a type II protein with the catalytic domain facing outside. It can also exist in an opposite type III orientation with its catalytic domain facing the cytosol [63, 86]. CD38 is observed in intracellular membranes, including endoplasmic reticulum, nucleus, mitochondria, and endolysosome [63, 87–90]. It catalyzes the synthesis of ADPR and cADPR using NAD^+^ as a substrate and is an important regulator of extracellular and intracellular NAD^+^ pools [91]. Both ADPR and cADPR act as second messengers controlling multiple cell functions through inducing intracellular Ca^2+^ fluxes independent of IP3 [92]. CD38 has been reported to play a role in SC differentiation [93]. For example, the NAD^+^/CD38/cADPR/Ca^2+^ signaling pathway antagonizes the cardiomyocyte differentiation of mouse ESCs [94].

Remarkably, increasing CD38 expression leads to a decline of NAD metabolites and distorts other NAD^+^-consuming enzyme activities in aged mice [91]. CD38 but not SIRT1 or PARPs is considered the predominant NAD^+^ consumers [95]. Therefore, it should also be considered whether CD38 has a specific regulatory role in SC senescence.

### 3.2. CD157

CD157 is a glycophosphatidylinositol-anchored protein. The ADP-ribosyl cyclase activity of CD157 is weaker than that of CD38. CD157 may participate in the embryonic and adult nervous systems partially through cADPR production [96]. CD157 upregulation increases the biosynthesis and transition of mitochondria from BMSCs to injured neurons, thus improves the neuroregeneration and inhibits cell apoptosis *via* calcium-dependent CD157/cyclic ADP-ribose pathway [97]. In addition, CD157 is a marker of tissue-resident vascular endothelial SCs (VESCs) [98, 99]. The CD157-positive endothelial cells have SC properties, including homeostatic capillary maintenance and regenerative capacity after vascular injury *in vivo* [100]. However, whether the functions of CD157 as a cell receptor are relevant to NAD^+^ metabolism remains unclear [3].

### 3.3. SARM1

The Toll/interleukin receptor (TIR) domain is necessary for SARM1 activity. Dimerization of TIR domain cleaves NAD^+^ into ADP-ribose, cADPR, and NAM [7, 101]. In addition to its involvement in innate immunity [102, 103], SARM1 is thought to an important NAD^+^-consuming enzyme during axonal injury in neurons [1, 104, 105]. Inhibition of SARM1 activation may be a compelling therapeutic target to treat neurodegenerative diseases [106–108]. However, the role of SARM1 in regulating SC homeostasis is currently unclear. Our RNA-Seq results (SRA: SRP152900) revealed significantly higher levels of *SARM1* expression in differentiated limbal SCs (LSCs) compared to undifferentiated ones. This suggests that maintaining SC stemness may also require inhibition of SARM1 activation.

Moreover, one-carbon metabolism enzyme methylenetetrahydrofolate dehydrogenase 2 (MTHFD2) has been reported to use NAD^+^ as a cofactor [109]. It is a dual-function factor for determining the pluripotency of pluripotent SCs through both preventing mitochondrial dysfunction and promoting homologous recombination repair in nucleus [110]. NAD^+^ can also be consumed by NAD^+^ kinase (NADK) to increase NADP^+^ production [111]. Through CD38, NADP^+^ is further converted into NAADP, which is another Ca^2+^-mobilizing second messenger [112]. Unfortunately, there is no report on crosstalk between NADK and CD38 in the regulation of SC homeostasis.

## 4. Conclusion

The NAD^+^ consumers systematically reduce the pool of NAD^+^ available for NAD^+^-specific enzymes and processes [11, 13]. These different enzymes can also interplay with each other to perform the same cellular function (Table 2). For instance, aging triggers NAD^+^ loss because of PARP activation in old mice, and NAD^+^ reduction lowers the activity of the antiaging proteins, sirtuins, leading to a feedforward cycle of aging [8, 113]. Another study found that CD38 has a central role in age-related NAD^+^ decline. CD38 inhibitor 78c upregulates NAD^+^ levels, thereby activating prolongevity and health span-related factors, including sirtuins and PARPs [114]. Moreover, PARP inhibitor PJ34 preserved intracellular NAD^+^ levels, increased SIRT1 activity, and improved the function in aging-induced endothelial progenitor cells (EPCs) [115]. In CSCs, NAMPT regulates epithelial-mesenchymal transition (EMT) and tumor dedifferentiation/reprograming *via* controlling cellular functions that promote proliferation and pathways mediated by SIRT1 and PARP1 [116]. Above results suggest that NAD^+^ availability can be achieved by inhibiting one or more of the NAD^+^ consumers to crosstalk the regulatory networks. Of course, SC functions might be restored or improved by supplying NAD^+^ precursors for biosynthesis [17, 50], including nicotinamide riboside (NR) [8, 117–119], NAM [120], and nicotinamide mononucleotide (NMN) (Table 3) [121]. This may be a potential approach for maintaining SC homeostasis and is a challenge for clinical treatment of related diseases.

## Figures and Tables

**Table 1:** Sirtuin functions for stem cell homeostasis.

Sirtuins	Stem cells	Targets	Functions	References
SIRT1	MSC	PGC-1*α*, TFAM, PARP1, FOXO1, FOXO3	Promoting mitochondrial fitness, DNA repair, and other aging-associated pathways	[17]
	MuSC	MYLK2, MYOG	Maintaining stemness and homeostasis	[8]
		UPR^mt^	Maintaining mitochondrial homeostasis and self-renewal	[14]
	NSC	FOXO3	Control over the circadian clock, limit exhaustion of their population	[19]
	Mouse ESC	MAT2a, SMPDL3B	Promoting pluripotency and embryogenesis	[15, 16]
	Rat MSC	NAMPT	Postponing senescence	[18]
	Liver CSC	*β*-Catenin/NANOG, SOX2	Maintaining self-renewal	[20, 21]

SIRT2	MuSC	PAX7	Promoting function and differentiation	[24]
	HSC	NLRP3	Preventing mitochondrial stress-induced cell death	[25]
		ALDOA, GAPDH, PGK1, ENO1	Regulating the metabolic transition	[26]

SIRT3	HSC	Antioxidant enzymes	Maintaining homeostasis	[29]
	HSC	Antiaging genes	Delaying senescence	[30]
	NSC	Apoptosis-related proteins	Ameliorating microglia activation-induced oxidative stress injury	[37]
	MSC	Antioxidant enzymes	Counteracting senescence, restoring their differentiation capacity, and reduces oxidative stress	[31, 32]
	MSC	laminB1, KAP1 and HP1*α*, etc.	Counteracting senescence	[34]
	SSC	Aerobic respiration-related factors	Promoting differentiation	[38, 39]
	Mouse MSC	Antioxidant enzymes	Counteracting senescence	[33]
	Rat MSC	PGC-1*α*/SIRT3/HIF-1*α*	Regulating mitochondrial quality and function	[35]
	Mouse ESC	TAC-related enzymes	Promoting *β* cell maturation	[36]

SIRT4	TSC	LSD1	Positive effect on senescence	[41]
	Breast CSC	SIRT1, H4K16ac, BRCA1	Negative effect on self-renewal	[42]

SIRT5	ADMSC	TCA-related enzymes	Accelerating senescence	[48]
	MEF	IDH2, G6PD	Enhancing cellular antioxidant defense	[50]

SIRT6	iPSC	Pluripotent genes	Differentiating into EBs and cardiomyocytes	[54]
	MSC	NRF2	Maintaining homeostasis	[58]
	HSC	Wnt target genes	Maintaining self-renewal	[53]

SIRT7	HSC, MSC	NRF1	Regulating cellular energy metabolism, proliferation, and regenerative capacity	[63, 64]
	HFSC	NFATc1	Initiating cell cycle	[65]
	MSC	*β*-Catenin, AXIN	Inhibiting osteogenic differentiation	[66]

ADMSC: adipose-derived mesenchymal stem cell; ALDOA: aldolase A; BRCA1: breast cancer susceptibility gene 1; CSC: cancer stem cells; EBs: embryoid bodies; ENO1: enolase 1; ESC: embryonic stem cell; FOXO1: forkhead box O1; FOXO3: forkhead box O3; GAPDH: glyceraldehyde-3-phosphate dehydrogenase; G6PD: glucose-6-phosphate dehydrogenase; HFSC: hair follicle stem cell; HIF-1*α*: hypoxia-inducible factor-1alpha; HP1*α*: heterochromatin protein 1alpha; HSC: hematopoietic stem cell; H4K16ac: acetylation of lysine 16 on histone H4; IDH2: isocitrate dehydrogenase-2; iPSC: induced pluripotent stem cell; KAP1: KRAB domain-associated protein 1; LSD1: lysine-specific demethylase 1; MAT2a: methionine adenosyltransferase 2a; MEFs: murine embryonic fibroblast; MSC: mesenchymal stem cell; MuSC: muscle stem cell; MYLK2: myosin light chain kinase 2; MYOG: myogenin; NAMPT: nicotinamide phosphoribosyltransferase; NFATc1: nuclear factor of activated T cells c1; NLRP3: nucleotide-binding domain, leucine-rich-containing family, pyrin domain-containing-3; NRF1: nuclear factor erythroid 2-related factor 1; NRF2: nuclear factor erythroid 2-related factor 2; NSC: neural stem cell; PARP1: poly(ADP-ribose) polymerase 1; PAX7: paired box 7; PGC-1*α*: peroxisome proliferator-activated receptor-gamma coactivator-1alpha; PGK1: phosphoglycerate kinase 1; SIRTs: sirtuins; SMPDL3B: sphingomyelin phosphodiesterase acid-like 3B; SSC: spermatogonial stem cell; TCA: tricarboxylic acid cycle; TFAM: mitochondrial transcription factor A; TSC: trophoblast stem cell; UPR^mt^: mitochondrial unfolded protein response.

**Table 2:** Functions of NAD^+^-consuming enzymes in stem cells.

Functions	NAD^+^-consuming enzymes
Stemness and homeostasis	SIRT1^↑^, SIRT3^↑^, SIRT6^↑^, PARP1^↑^, CD157^↑^, MTHFD2^↑^
Self-renewal	SIRT1^↑^, SIRT4^↓^, SIRT6^↑^, PARP1^↑^
Mitochondrial fitness	SIRT1^↑^, SIRT2^↑^, SIRT3^↑^, SIRT5^↑^, CD157^↑^
Senescence	SIRT1^↓^, SIRT3^↓^, SIRT4^↑^, SIRT5^↑^, SIRT7^↓^, CD38^↑^
Differentiation	SIRT2^↑^, SIRT3^↑^, SIRT6^↑^, SIRT7^↓^, PARP1^↑^, CD38^↑^, SARM1^↑^?, NADK^↑^?
Metabolic regulation	SIRT2, SIRT7, CD38

↑: positive effect on the function; ↓: negative effect on the function; MTHFD2: methylenetetrahydrofolate dehydrogenase 2; NADK: NAD^+^ kinase; PARP: poly(ADP-ribose) polymerase; SARM1: sterile alpha and Toll/interleukin receptor (TIR) motif-containing 1; SIRT: sirtuin.

**Table 3:** Improvement of stem cell homeostasis by adding NAD^+^ precursors.

NAD^+^ precursors	Concentrations	Stem cells	Functions	References
NR	400 mg/kg/day	MuSC	Antiaging	[8]
	500 mg/kg/day	ISC	Antiaging	[117]
	500 *μ*M	iPSC	Ameliorating mitochondrial function	[118]
	400 mg/kg/day	HSC	Restoring youthful metabolic capacity	[119]

NAM	10 mM	hESC	Promoting pancreatic differentiation	[120]

NMN	0.03–2.25 *μ*M	MSC	Maintaining self-renewal	[121]

hESC: human embryonic stem cell; HSC: hematopoietic stem cell; iPSC: induced pluripotent stem cell; ISC: intestinal stem cell; MSC: mesenchymal stromal cell; MuSC: muscle stem cell; NAM: nicotinamide; NMN: nicotinamide mononucleotide; NR: nicotinamide riboside.

## Data Availability

Our RNA-Seq data supporting this review have been deposited in the Sequence Read Archive (SRA) database at NCBI under the accession number SRP152900. The raw data are available (https://www.ncbi.nlm.nih.gov/sra/SRP152900).

## Abbreviations

ADMSC: Adipose-derived mesenchymal stem cell
ADP: Adenosine diphosphate
ADPR: ADP-ribose
ALDOA: Aldolase A
cADPR: Cyclic ADPR
CAT: Catalase
CSC: Cancer stem cells
EB: Embryoid body
EPC: Endothelial progenitor cell
EMT: Epithelial-mesenchymal transition
ENO1: Enolase 1
FGF4: Fibroblast growth factor 4
FOXO3a: Forkhead box O3a
GAPDH: Glyceraldehyde-3-phosphate dehydrogenase
HFSC: Hair follicle stem cell
HSC: Hematopoietic stem cell
iPSC: Induced pluripotent stem cell
LSC: Limbal stem cell
MAT2a: Methionine adenosyltransferase 2a
MEF: Murine embryonic fibroblast
mESC: Mouse embryonic stem cell
MTHFD2: Methylenetetrahydrofolate dehydrogenase 2
MuSC: Muscle stem cell
MnSOD: Manganese superoxide dismutase
NAD^+^: Nicotinamide adenine dinucleotide
NADK: NAD^+^ kinase
NAM: Nicotinamide
NAMPT: Nicotinamide phosphoribosyltransferase
NFATc1: Nuclear factor of activated T cells c1
NLRP3: Nucleotide-binding domain, leucine-rich-containing family, pyrin domain-containing-3
NPC: Neural progenitor cell
NRF1: Nuclear factor erythroid 2-related factor 1
NRF2: Nuclear factor erythroid 2-related factor 2
NMN: Nicotinamide mononucleotide
NR: Nicotinamide riboside
NSC: Neural stem cell
PARP: Poly(ADP-ribose) polymerase
PAX7: Paired box 7
PDGFR*α*: Platelet-derived growth factor receptor *α*
PGK1: Phosphoglycerate kinase 1
ROS: Reactive oxygen species
SARM1: Sterile alpha and Toll/interleukin receptor (TIR) motif-containing 1
SC: Stem cell
SIRT: Sirtuin
SMPDL3B: Sphingomyelin phosphodiesterase acid-like 3B
SOX2: SRY-box transcription factor 2
SSC: Spermatogonial stem cell
TET2: Ten-eleven translocation-2
TIR: Toll/interleukin receptor
TSC: Trophoblast stem cell
UPR^mt^: Mitochondrial unfolded protein response
VESC: Vascular endothelial stem cell.

## Supplementary Materials

Supplementary Materials Supplementary Figure S1: literature search method and selection criteria. Searches in Web of Science and PubMed were restricted to papers published between 2016 and 2022. The basic framework of the intervention program was determined by searching for a combination of subject terms and free words, such as “stem cell,” “NAD enzyme,” and “homeostasis.” Exclusion criteria for literature are as follows: no complete text accessible; publications written in a language other than English; and studies with inadequate literature evaluation.
