# Supplementary material for: NAD+-Consuming Enzymes in Stem Cell Homeostasis
Source: Oxid Med Cell Longev. 2023 Feb 8;2023:4985726. doi: 10.1155/2023/4985726 (PMC9931471; doi:10.1155/2023/4985726)
Supplement: Supplementary Materials — Supplementary Figure S1: literature search method and selection criteria. Searches in Web of Science and PubMed were restricted to papers published between 2016 and 2022. The basic framework of the intervention program was determined by searching for a combination of subject terms and free words, such as “stem cell,” “NAD enzyme,” and “homeostasis.” Exclusion criteria for literature are as follows: no complete text accessible; publications written in a language other than English; and studies with inadequate literature evaluation. [file 4985726.f1.pdf]

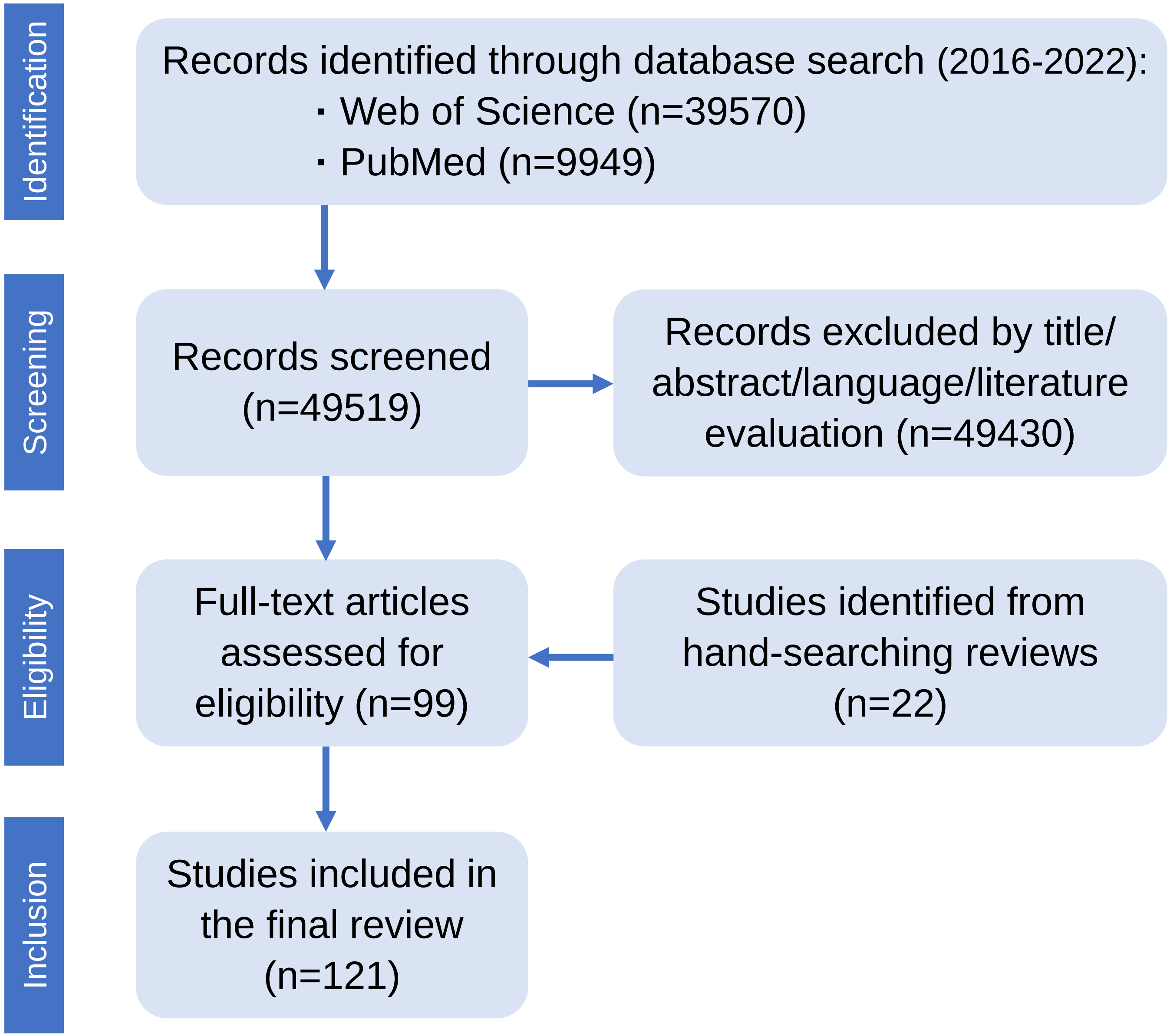
Identification
Records identified through database search (2016-2022):
▪ Web of Science (n=39570)
▪ PubMed (n=9949)
Screening
Records screened (n=49519)
Records excluded by title/ abstract/language/literature evaluation (n=49430)
Eligibility
Full-text articles assessed for eligibility (n=99)
Studies identified from hand-searching reviews (n=22)
Inclusion
Studies included in the final review (n=121)
